# Transcoronary Sinus Therapy for Coronary Microvascular Dysfunction

**DOI:** 10.31083/RCM38565

**Published:** 2025-08-19

**Authors:** Jingjing Rao, Yue Wang, Lina Tan, Liangbo Hu, Xiaocong Zeng

**Affiliations:** ^1^Department of Cardiology, The First Affiliated Hospital of Guangxi Medical University, 530021 Nanning, Guangxi, China; ^2^Guangxi Key Laboratory Base of Precision Medicine in Cardiocerebrovascular Diseases Control and Prevention & Guangxi Clinical Research Center for Cardio-Cerebrovascular Diseases, 530021 Nanning, Guangxi, China; ^3^School of Basic Medical Sciences, Guangxi Medical University, 530021 Nanning, Guangxi, China

**Keywords:** coronary microcirculatory disorders, coronary sinus reducer, pressure-controlled intermittent coronary sinus occlusion, angina pectoris, ST-segment elevation myocardial infarction

## Abstract

Coronary microvascular disease has been found to increase the incidence of the composite endpoint for cardiovascular events and affect coronary revascularization. Coronary microvascular disease is often accompanied by epicardial disease, and despite successful revascularization and optimal medications, coronary microvascular disease may lead to reduced exercise tolerance and worsening clinical symptoms. Moreover, despite advances in percutaneous coronary intervention for coronary revascularization, the management of microvascular obstruction in reperfused myocardial tissue remains challenging and is a high-risk procedure. Previous studies have identified the coronary venous system as a new avenue for treating coronary microvascular obstructions associated with revascularization. Current data suggest that coronary sinus interventions, which primarily include coronary sinus reducer and pressure-controlled intermittent coronary sinus occlusion interventions, can provide significant clinical aid in 70–80% of patients with refractory angina pectoris and acute myocardial infarction who suffer from microvascular disease with no possibility of revascularization by modulating coronary venous pressures. However, a recent randomized trial demonstrated no difference in infarct size reduction between the pressure-controlled intermittent coronary sinus occlusion-assisted and conventional primary percutaneous coronary intervention groups. This article reviews recent advancements in coronary sinus-based therapeutic approaches for coronary microvascular disease.

## 1. Introduction

Since the early 20th century, cardiovascular diseases have been the leading cause of disease-related mortality in developed countries [1]. Among these, ischemic heart disease remains the primary contributor to premature mortality and disability-adjusted life years globally [2]. Coronary microvascular dysfunction (CMD) is increasingly recognized as a pathophysiologically relevant mechanism in ischemic heart disease [3], demonstrating high prevalence among patients with extensive cardiovascular risk factors and correlating with elevated risks of adverse clinical outcomes [4]. Coronary circulation is a complex system consisting of three vascular segments: anterior small arterioles, small arterioles, and capillaries [5], which are the main resistance vessels in the coronary arteries and play a key role in regulating coronary artery perfusion pressure and physiologic regulation [6]. Under pathological conditions, such as atherosclerotic and non-atherosclerotic pathogenic factors, structural (microvascular remodeling, luminal obstruction, vascular invasion, capillary rarefaction, and perivascular fibrosis) [7, 8] and functional (endothelial cell dysfunction, microvascular spasm, and cardiac sympathetic neuron dysfunction) [4, 9, 10, 11, 12, 13, 14] abnormalities of the coronary microcirculation lead to coronary artery microvascular dysfunction. CMD has been found to increase the incidence of the composite endpoint of cardiovascular events, which may contribute to the pathophysiology of cardiovascular death and heart failure and affect coronary revascularization [15]. The main manifestation of coronary microvascular dysfunction associated with hemodialysis is the absence of reflow [16]. Additionally, CMD is often accompanied by epicardial disease, which may lead to reduced exercise tolerance and worsening of clinical symptoms even with successful revascularization and optimal medication (OMT). Despite advancements in direct percutaneous intervention for coronary revascularization, the management of microvascular obstruction in reperfused myocardial tissue remains challenging and is a high-risk procedure [17, 18]. Study has demonstrated a significant increase in long-term major adverse cardiovascular events (MACE) in patients with post-procedural combined coronary microcirculatory obstruction during elective percutaneous coronary intervention (PCI) [16]. This has generated interest in the coronary venous system as an alternative route for treating coronary microvascular disorders associated with hemodialysis. Coronary venous sinus intervention can positively modulate coronary microvascular function. This review focuses on the main approaches for treating coronary microvascular disorders via the coronary venous sinus.

## 2. Coronary Venous Sinus Characteristics and Development in Myocardial Ischemia 

### 2.1 Anatomical Basis

The unique characteristics of the coronary venous sinus make it a viable therapeutic target for coronary ischemia. One main reason for this is that the coronary venous sinus is the most constant feature of the cardiac venous system. It is a tubular venous structure located in the lower part of the left atrioventricular groove. The coronary venous sinus is the largest of the cardiac veins, with a diameter of up to 12 mm and a length ranging from 30 to 63 mm, making it readily accessible in most patients [19, 20]. The coronary sinus (CS) receives blood from several sources, including the posterior left ventricular vein and the posterior left atrial vein, in addition to the greater cardiac vein, the middle cardiac vein, and the lesser cardiac vein [19, 21]. Most of the venous blood from the heart drains back to the right atrium through the CS [22]. Additionally, the coronary venous vascular system is a dense meshwork of many interconnected vessels and is unaffected by the atherosclerotic disease process, providing an excellent anatomical basis for the treatment of coronary ischemia by increasing CS pressure [23].

### 2.2 Early Surgical Approaches

As early as 1898, F.H. Pratt demonstrated the role of reverse perfusion of oxygenated blood in maintaining myocardial viability in animals [23]. Since then, many studies have investigated the treatment of coronary artery ischemia through CS intervention. The first transcoronary sinus intervention for coronary artery ischemia was performed by Beck *et al*. [24] in 1948, in which an anastomosis between the aorta and the CS was created, followed by partial sinus ligation to improve ischemia . Increasing coronary artery pressure improves coronary capillary and microvascular patency, redistributes blood perfusion, and reduces ischemia. In addition to De Maria’s study [25], the beneficial effects of CS arterialization have significantly diminished over time. In a 6-month animal series on CS arterialization, only an increase in intercoronary anastomotic blood flow was observed; however, demonstrating any significant reversal of perfusion in the myocardial capillary bed was not possible [26]. Although this procedure improves myocardial ischemia and prevents ventricular fibrillation, it causes intramyocardial hemorrhage and may be associated with high long-term mortality [27]. Another disadvantage of this procedure is the permanent reduction in coronary venous drainage and altered ultrastructural changes in the CS wall [27, 28].

### 2.3 Transition to Catheter-Based Interventions

Compared with the aforementioned complex and time-consuming techniques, CS catheter insertion offers the possibility of rapid access to the coronary microcirculation. Simultaneous retrograde perfusion for myocardial ischemia was proposed by Meerbaum as a treatment to enhance retrograde delivery of arterial vasculature to the acutely ischemic myocardium during diastole and promote coronary venous drainage during systole. The experiments were performed by acutely occluding the anterior descending branch of the canine left coronary artery for 75 min and establishing diastolic reverse perfusion for 45 min by synchronously pumping arterial blood from the brachiocephalic artery into the anterior interventricular coronary vein after the first 30 min of occlusion. Significant improvements in myocardial ischemia relief and local dysfunction were observed [29]. However, the advent of coronary artery bypass grafting and PCI led to the demise of surgical coronary artery arteriovenous bypass grafting in clinical practice, and its application remains limited [23, 30, 31, 32, 33]. Consequently, the coronary venous system has been increasingly studied over the last two decades and has been found to be an alternative approach for treating coronary microvascular disorders associated with blood flow reconstruction. The primary new approaches for treating coronary microvascular disorders through the CS include CS resurfacing and percutaneous pressure-controlled intermittent coronary sinus occlusion (PICSO) [22, 34, 35]. Both methods regulate intravascular blood by increasing coronary venous pressure, which redistributes blood from non-ischemic areas to ischemic areas of the myocardial tissue. The normal myocardium undergoes selective sympathetically mediated contraction of the subepicardial vasculature during exercise, and the subendocardium receives preferential perfusion; however, in patients with coronary artery disease (CAD), this compensatory mechanism fails. Thus, when the epicardial coronary arteries are stenosed, both subendocardial and subepicardial blood flow is reduced; however, the subendocardium is more susceptible to the effects of ischemia than the middle layer of the myocardium or the subepicardium [36]. Additionally, when myocardial ischemia is present, impaired myocardial contractility leads to elevated left ventricular end-diastolic pressure, which exerts external pressure on the subendocardial capillaries, increasing the resistance to blood flow to the subendocardium and exacerbating local ischemia. Elevated CS pressure increases the backward pressure in small veins and capillaries, resulting in a slight dilation of the capillary diameter and a significant decrease in resistance to flow. Owing to reduced subendocardial capillary resistance, the normal subepicardial-to-subendocardial flow ratio is restored; the main mechanism involves the establishment of a complementary mechanism, with elevated CS pressures, distal vasodilatation and high pressures in the vasculature causing pre-existing collateral connections to open up and new coronary collateral branches to be established over time [37]. Simultaneously, the increased back pressure in the precapillary small arterial system induces subendocardial capillary dilation, which distributes blood from the epicardium to the subendocardium, thus improving the degree of subendocardial ischemia in the ischemic region [37, 38].

## 3. Clinical Research Evidence for the Coronary Sinus Reducer

### 3.1 Device Design and Biomechanical Mechanism

**Fig. 1.**
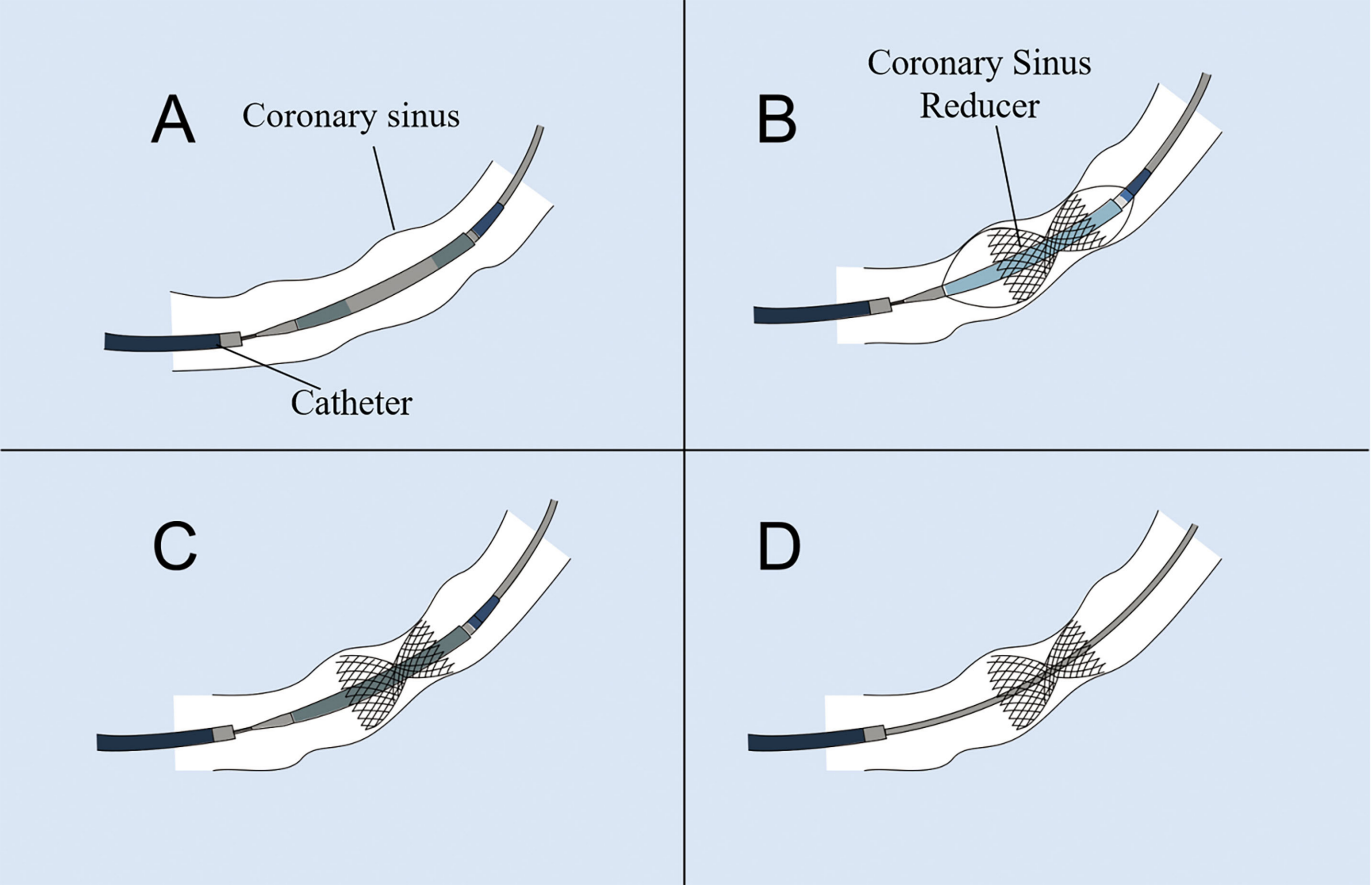
**Schematic illustration describing the implantation processof the coronary sinus (CS) reducer**. (A) The CS reducer is advanced over the guidewire and positioned at the CS. (B) The balloon is inflated to deploy and secure the CS reducer at the CS. (C) The balloon is deflated and retrieved. (D) The guidewire is withdrawn.

**Fig. 2.**
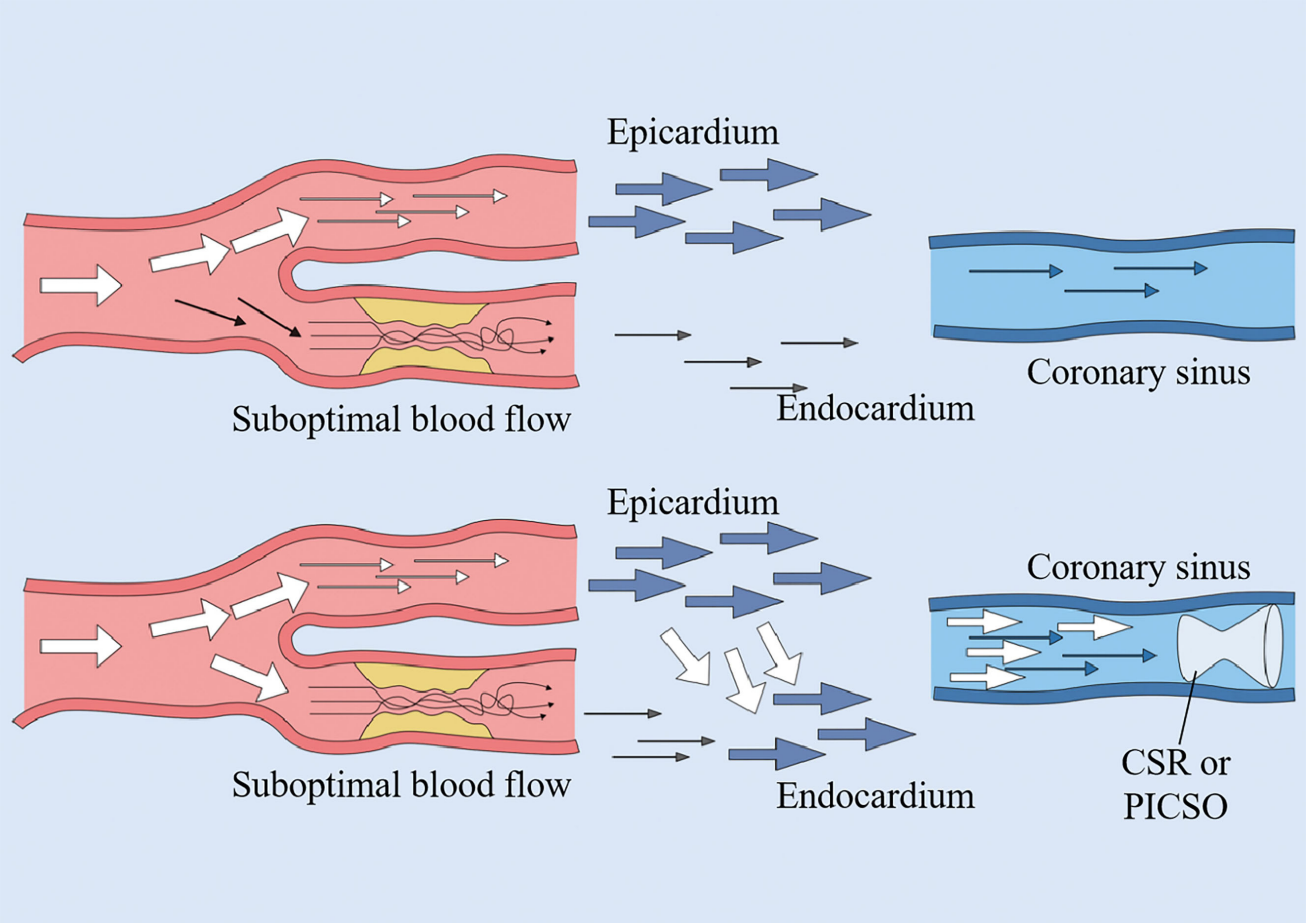
**Coronary sinus intervention mechanisms**. Both pressure-controlled intermittent coronary sinus occlusion (PICSO) and coronary sinus reducer (CSR) function by elevating coronary venous pressure. This hemodynamic modulation facilitates blood redistribution from the subepicardial to subendocardial myocardial layers, thereby redirecting perfusion from non-ischemic zones toward ischemic myocardial territories.

The CS reducer is a stainless-steel balloon-expandable stent, a percutaneous implantable device in the shape of an hourglass (Fig. 1). It has a fixed 3-mm diameter at the neck with diameters at the ends that can be adjusted up to 7–13 mm by pressurized filling. The stent is asymmetrical at both ends, with a proximal diameter larger than the distal end to accommodate the tapered anatomy of the CS, which results in stenosis of the CS, thus increasing the coronary venous pressure and redistributing the blood from the non-ischemic region to areas of the ischemic myocardial tissue [37, 39] (Fig. 2).

### 3.2 Clinical Efficacy in Refractory Angina Pectoris

The first human study on CS reducers was performed in 2007 [37]. In this non-randomized prospective study, a CS reducer was successfully implanted in the CS of 15 patients with refractory angina pectoris, and none experienced clinical complications or prognostic adverse events after the procedure. A significant improvement in angina scores was observed 6 months post-implantation, with the mean Canadian Cardiovascular Society (CCS) score decreasing from 3.07 to 1.64 in 14 patients (*p* < 0.0001). The mean loading dobutamine echocardiography score at 6 months decreased from 25.08 to 21.08 in 13 patients (*p* < 0.004) [37]. In two centers, CS reducers were implanted in 23 eligible patients with severe refractory angina pectoris, and angina severity and myocardial ischemia were evaluated 6 months after successful CS reducer implantation in 21. The CCS score was reduced from 3.3 to 2.0 at baseline (n = 20, *p* < 0.01), and the ventricular wall motion score index was also significantly improved (n = 8, 1.9 ± 0.4 vs. 1.4 ± 0.4, *p* = 0.046). CS reducers implantation is safe; however, questions regarding the placebo effect need to be addressed in more clinically randomized trials [40]. The CS reducer was further evaluated in a randomized, double-blind, sham-operated controlled, multicenter clinical trial (COSIRA) involving 104 patients with refractory angina and myocardial ischemia. The patients were randomly assigned to receive either CS reducer implantation or drug therapy. At 6 months, a greater proportion of patients in the device therapy group showed an improvement of one CCS grade (71% vs. 42%, *p* = 0.003) and two CCS grades (35% vs. 15%, *p* = 0.02) compared with the control group. Quality of life, as measured by the Seattle Angina Questionnaire score, improved in the device group compared with the control group (17.6 vs. 7.6 points, *p* = 0.048) [39]. Many studies have demonstrated that CS reset devices are effective in relieving angiogenic symptoms in patients with obstructive CAD who are not eligible for hemodialysis [41, 42, 43, 44, 45, 46, 47, 48]. The REDUCER-I study, a non-randomized, multicenter investigation conducted across 25 centers in 9 European countries, evaluated the CS Reducer therapy in patients with refractory angina. Among 371 patients who underwent successful CS reducer implantation, 361 (97%) were eligible for primary safety endpoint analysis at 6-month follow-up. At 6 months post-procedure, the mean CCS angina class score significantly decreased from 2.8 ± 0.6 at baseline to 1.8 ± 0.8 (*p* < 0.0001). Both the Seattle Angina Questionnaire Quality of Life (SAQ-QOL) total score and its angina stability and frequency subscales demonstrated significant improvements from baseline (all *p* < 0.0001) [49]. Table 1 (Ref. [37, 39, 40, 41, 42, 44, 47, 48, 49, 50, 51]) summarizes the results of CSR clinical studies in patients with refractory angina.

**Table 1.** **Clinical trial of Coronary sinus reducer in the treatment of refractory angina**.

Study (publication year)	Patient number	Follow-up time (month)	Response	CCS		*p*
				before	after	
Banai *et al*. (2007) [37]	14	6	12 (85.6%)	3.07	1.64	<0.0001
Verheye *et al*. (2015) [39]	52	6	37 (71.1%)	3.2 ± 0.4	2.1 ± 1.0	=0.001
Konigstein *et al*. (2014) [40]	20	6	17 (85%)	3.35 ± 0.6	2.0 ± 1	<0.001
Konigstein *et al*. (2018) [41]	39	6	33 (84.6%)	3.4 ± 0.5	2.0 ± 1	<0.001
Giannini *et al*. (2018) [42]	50	4	40 (80%)	2.98 ± 0.52	1.67 ± 0.83	<0.001
Ponticelli *et al*. (2019) [47]	44	24	34 (77.2%)	2.98 ± 0.5	1.74 ± 0.86	=0.001
Verheye *et al*. (2021) [44]	220	6	183 (83%)	2.8 ± 0.6	1.8 ± 0.7	<0.001
Ponticelli *et al*. (2021) [48]	599	16	455 (76%)	>3.0	<2	<0.001
Giannini *et al*. (2017) [50]	8	4	7 (87.5%)	3.0	1.5	<0.014
Verheye *et al*. (2024) [49]	344	6	240 (70%)	2.8 ± 0.6	1.8 ± 0.8	<0.0001
Tebaldi *et al*. (2024) [51]	21	4	16 (76.1%)	>3	<2	<0.001

CCS, Canadian Cardiovascular Society. Response: The number of patients with an improvement of ≥1 grade in the Canadian Cardiovascular Society score.

### 3.3 Therapeutic Effects on Microvascular Dysfunction

However, in patients with other chronic heart diseases characterized by angina and subendocardial ischemia such as microvascular angina pectoris, further investigation is necessary to determine whether this treatment may also be beneficial in obstructive CAD. Coronary microvascular dysfunction appears to be the underlying pathophysiological mechanism in patients with refractory angina and evidence of myocardial ischemia. Several studies have demonstrated a positive impact of CS reducer in patients with refractory symptoms secondary to coronary microcirculatory disorders [50, 51]. However, data regarding their effect on coronary microvascular function are lacking. Pagnesi first used the CS decompensator system to treat patients with refractory angina pectoris and non-obstructive CAD. Patients suffering from chronic stable angina pectoris (CCS grades 3–4) who had noninvasive myocardial ischemia despite OMT were screened. Implantation of a CS reducer resulted in a decrease in median CCS classification from 3.0 to 1.5 (*p* < 0.014), significant improvement in most questionnaire domains of the Seattle Angina Questionnaire, and an increase in quality of life score from 26.5 to 56.0 (*p* < 0.018) after 1 year of follow-up. Myocardial Perfusion Reserve Index of the ischemic segments significantly increased after reducer implantation in all three patients (*p* < 0.001). The CS reducer is safe and has a unique sustained biological effect of normalizing the ratio of subendocardial to subepicardial blood flow [50]. Recently, Tebaldi *et al*. [51] conducted the INROAD study (Index for the Evaluation of Microcirculatory Resistance in Patients with Implanted Coronary Sinus Tapering Tubes), in which 24 patients with obstructive CAD and previous coronary revascularization treated with tapering tube implantation underwent repeat invasive coronary physiologic assessments 4 months after successful implantation of CS reducers in 21 patients. Microcirculatory resistance index values decreased from 33.35 ± 19.88 at baseline to 15.42 ± 11.36 (*p* < 0.001). Significant reductions (≥20% from baseline) in the microcirculatory resistance index were observed in 15 patients. The number of patients with an abnormal (≥25) microcirculatory resistance index decreased from 12 to 4 (*p* = 0.016) [51]. Although the study demonstrated high statistical power for the primary endpoint, the sample size was limited. Therefore, larger-scale investigations are required to validate these findings.

### 3.4 Issues and Future Research Directions

These findings suggest that CS reducer implantation significantly improves the functional parameters of coronary microcirculation and may be effective in the treatment of CMD. Although the results of this study showed that CS reducers have a positive role in the treatment of coronary microcirculatory disorders, 30% of patients still did not improve. Therefore, the treatment of CMD using CS reducers warrants further research to ensure the continued success and effectiveness of this technique. Several prospective trials are currently underway, and the results of a randomized trial comparing CS reducer with pharmacotherapy in patients with microvascular dysfunction (COSIMA [coronary sinus reducers for the treatment of refractory microvascular angina]; NCT 04606459) are promising.In addition, the evaluation of microvascular function through ongoing clinical trials such as the ​​CO​​ronary ​​S​​Inus ​​R​​educer for ​​R​​efractory ​​A​​ngina ​​II​​ (COSIRA-II) trial (NCT05102019)—a randomized controlled trial assessing the efficacy of the Coronary Sinus Reducer in patients with refractory angina type II—and the ​​RE​​discovery of ​​MED​​ical Therap​​Y​​ in ​​P​​atients with ​​I​​schemia and ​​L​​ow-​​O​​bstructive Coronary Artery Disease (REMEDY-PILOT) study (NCT05492110) investigating Coronary Sinus Reducer implantation in patients with ischemia and non-obstructive coronary arteries or coronary microvascular dysfunction, is anticipated to provide additional mechanistic insights.

## 4. Evidence From Clinical Studies on PICSO

### 4.1 Technical Principles and Hemodynamic Mechanisms of PICSO 

**Fig. 3.**
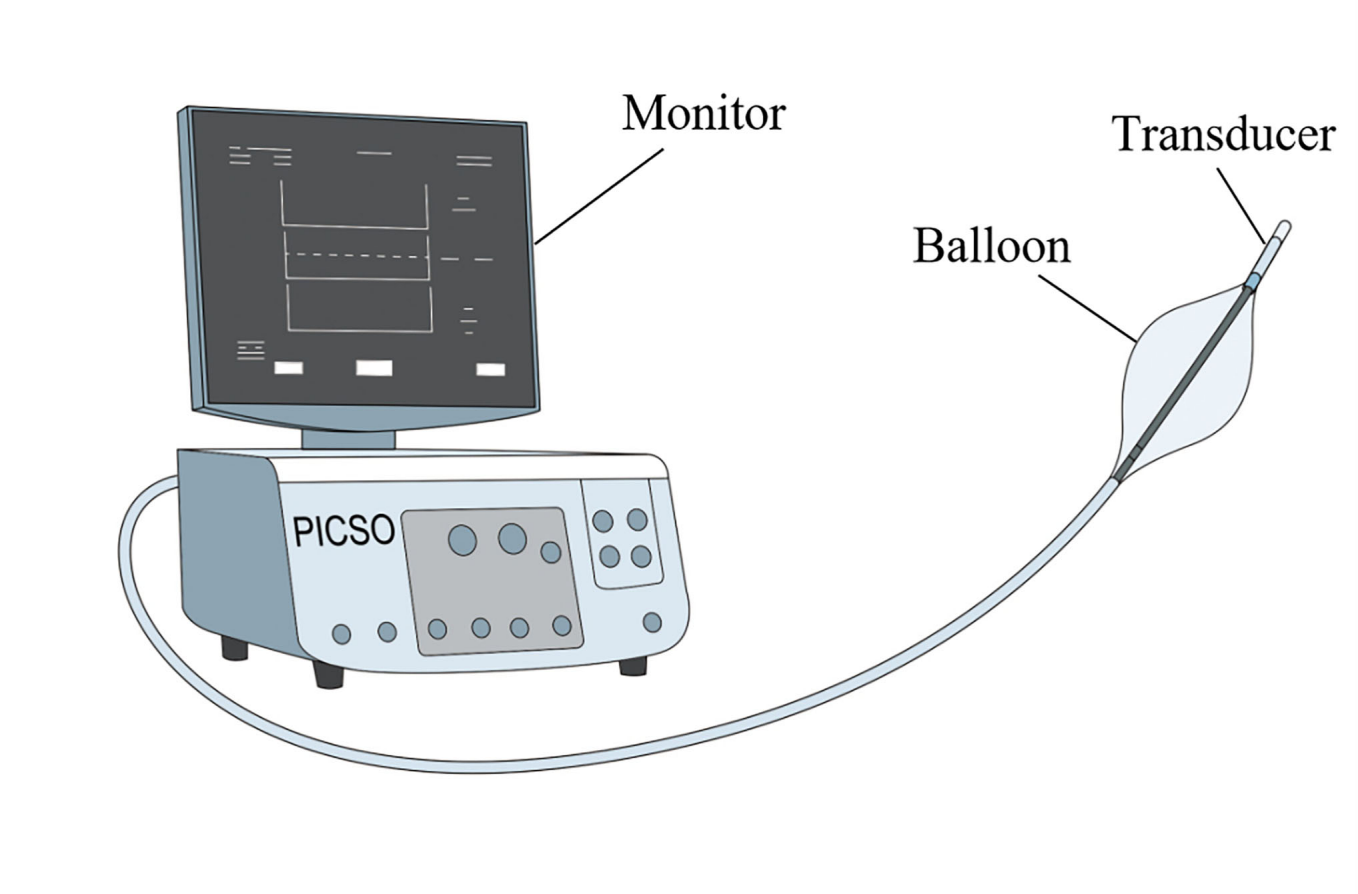
**Schematic diagram of the pressure-controlled intermittent coronary sinus occlusion (PICSO) device​**.

PICSO involves placing a balloon head-end catheter with a transducer for CS blood pressure monitoring at the CS orifice, leading to an increase in CS pressure (Fig. 3). Upon reaching a pressure plateau, the balloon is automatically retracted, thus generating pressure and flow pulsations that cause redistribution of blood flow within the coronary venous system and facilitate the distribution of blood to the edges of the ischemic myocardium [22, 52] (Fig. 2). PICSO induces a sustained increase and decrease in the pressure gradient within the microcirculatory bed, allowing the removal of toxic waste from the microcirculation [23, 53] in addition to inducing the release of vascular growth factors from the venous endothelium [54, 55], thus effectively reducing the infarct size and facilitating myocardial recovery after coronary artery occlusion [56, 57].

### 4.2 Preclinical Evidence Framework

Mohl hypothesized that longer intermittent CS occlusion using CS pressure measurements as a feedback guide for the duration of CS occlusion would be more effective, a technique known as PICSO. Intermittent CS occlusion was performed in a canine treatment group (n = 13) and a control group (n = 12), whose infarct size was measured at 6 h postoperatively. The myocardial infarction (MI) size of the treatment group was significantly smaller than that of the control group [58]. The effects of PICSO on myocardial ischemia were explored in domestic pigs. Artificial stenosis was induced in the left anterior descending coronary artery, reducing the lumen diameter by 80%. This significantly decreased blood flow in the intima and transmural layers distal to the stenosis compared with no stenosis (*p* < 0.01). Hemodynamics, local myocardial blood flow, and oxygen, lactate, and nucleoside metabolism were measured in animals after PICSO treatment. The results showed that PICSO did not alter the final level of myocardial ischemia but accelerated the rate of myocardial ischemic regression [57].

### 4.3 Clinical Efficacy and Safety Validation

In a randomized trial of 30 patients undergoing bypass surgery, PICSO was applied during early reperfusion for 1 h, and myocardial function was determined using short-axis cross-sectional views from intraoperative two-dimensional (2D) echocardiography. The preservation of motion-reduced segments in the PICSO-treated group was superior to preservation over the control group (–1.3 ± 2.4 vs. –9.1 ± 2.6 % fractional area change; *p* < 0.04). Additionally, metabolite elution during PICSO was superior to that of the control group. The findings suggest that PICSO is a safe procedure and that its short-term beneficial effects on myocardial function indicate protection of myocardial viability; however, the long-term effects of PICSO remain uncertain [59]. Another clinical trial involving 30 patients undergoing coronary artery bypass grafting showed that the application of PICSO was feasible and safe. The study also identified a variable in venous occlusion pressure that can be used in the closed-loop control system for this intervention and to evaluate the diagnostic volume for further optimization of PICSO [60]. This technique is gradually being applied in the treatment of acute ST-segment elevation MI (STEMI). In a prospective, multicenter, non-randomized study of 30 patients were successfully treated with primary PCI (pPCI) for anterior wall STEMI, 19 (63%) underwent PICSO, which was sustained for 90 (±2) min in 12 patients (40%). Patients were observed for infarct size from 2–5 days to 4 months post-treatment, and infarct size reduction was greater in patients successfully treated with PICSO than in matched controls (41.6 ± 8.2% vs. 27.7 ± 9.9%, respectively; *p* = 0.04) [61]. Further, pPCI + PICSO (initiated after reperfusion) for the treatment of patients with STEMI on day 5 showed a significant improvement in infarct size measured by cardiac magnetic resonance (CMR) [62]. The role of PICSO in patients with inferior wall STEMI due to right coronary artery occlusion was explored for the first time in humans in 2021 [63]. Thirty-six patients with STEMI (27 anterior and 9 inferior walls) underwent PICSO-assisted direct percutaneous intervention (PPCI) and were compared with a matched control group (n = 72) who underwent standard PCI. At 48 h and 6-month follow-up, the improvements in the index of microcirculatory resistance (IMR), resistance reserve ratio, infarct size, and microvascular obstruction were statistically different. PICSO treatment improves microvascular function and vasodilatation capacity and helps to reduce infarct size in patients with STEMI [63]. This study demonstrated that PICSO is safe and feasible for the treatment of STEMI. Pappalardo reported the first prolongation of PICSO therapy in two patients with refractory left ventricular (LV) dysfunction and persistent ischemia, resulting in significant improvements in myocardial ischemia and recovery of LV systolic function in both patients [64]. To evaluate the long-term outcomes of PICSO in patients with acute MI and revascularization, 34 patients with STEMI treated with or without PICSO were reanalyzed. Significant differences were observed in reinfarction (*p* = 0.015) and major adverse cardiovascular events (*p* < 0.0001) between the two groups. This study suggests that PICSO helps to reduce infarct size and can significantly reduce MACE during long-term follow-up [35]. Table 2 (Ref. [35, 59, 61, 62, 63, 65, 66]) summarizes the results of PiCSO clinical studies in patients with STEMI.

**Table 2.** **Clinical trial of PICSO in the treatment of STEMI**.

Study (publication year)	Indication	Design	Patient (control/PICSO)	Outcomes
Mohl *et al*. (1988) [59]	Undergoing coronary artery bypass grafting	PICSO was started after aortic declamping and continued for one hour during the early reperfusion phase.	15/15	Hypo-kinetic segments were preserved better in PICSO-treated patients than in controls, washout of metabolites during PICSO.
Mohl *et al*. (2008) [35]	STEMI	Balloon inflation for 10 s and deflation or 5 s were repeated, CS pressure was monitored continuously and gas volume in the CS balloon was optimized not to exceed 50 mmHg.	17/17	The PICSO group showed significantly less total CK release than that of the control group. PISCO group had significantly smaller abnormally contracting segments than the control group.
van de Hoef *et al*. (2015) [61]	Anterior STEMI	PICSO treatment delivered for 90 minutes.	13/19	PICSO was safe in the setting of STEMI and showed greater infarct size reduction between 2 and 5 days and 4 months compared to matched controls.
De Maria *et al*. (2018) [65]	Anterior STEMI	PICSO treatment was delivered to patients, until a minimum PICSO dose of 800 mmHg was achieved.	50/25	Compared to controls, patients treated with PICSO had a lower IMR at 24–48 hours and lower IS at six months.
Egred *et al*. (2020) [62]	Anterior STEMI	PICSO quantity of 800 mmHg was reached.	80/45	Infarct size at day 5 was significantly lower in the PICSO group, no MACE related to the PICSO intervention.
Scarsini *et al*. (2022) [63]	(27 anterior and 9 inferior) with STEMI	PICSO treatment was delivered for a minimum of 20 minutes until a PICSO dose of 800 mmHg was achieved.	72/36	IMR and RRR improved significantly in PICSO-treated patients compared with controls in patients. Patients treated with PICSO presented significantly less frequently with MVO and smaller 6-month IS compared with controls.
De Maria *et al*. (2024) [66]	Anterior STEMI	PICSO treatment was planned to be used and was initiated and maintained for at least 20 minutes. The optimal goal treatment time was defined as 45 ± 5 minutes.	73/72	No differences were observed in IS at 5 days and 6 months, nor were differences between PICSO-treated and control patients noted in terms of the occurrence of microvascular obstruction or intramyocardial hemorrhage. PICSO showed no increase in adverse events over a 6-month period.

PICSO, pressure-controlled intermittent coronary sinus occlusion; MACE, major adverse cardiac events; STEMI, ST-elevation myocardial infarction; IMR, index of microcirculatory resistance; IS, Infarct size; RRR, resistive reserve ratio; MVO, microvascular obstruction; CS, coronary sinus; CK, creatine kinase.

### 4.4 Current Controversies and Future Optimization Directions​

A recent randomized trial evaluated the effectiveness of PICSO therapy in patients with anterior wall STEMI. A total of 145 patients with anterior wall STEMI were equally randomized to the PPCI and conventional pPCI groups. No difference in infarct size between the two groups was observed at 5 days and 6 months postoperatively, respectively, at 5 days (27.2% ± 12.4% vs. 28.3% ± 11.45%; *p* = 0.59) and 6 months (19.2% ± 10.1% vs. 18.8% ± 7.7%; *p* = 0.83). Similarly, no significant difference was observed between the PICSO-treated group and the control group in the incidence of microvascular occlusion (67.2% vs. 64.6%; *p* = 0.85) or myocardial intracardiac hemorrhage (55.7% vs. 60%; *p* = 0.72). In this randomized trial, the procedure time and amount of contrast used were higher for PICSO than for conventional pPCI; however, no adverse events related to this device were reported over the 6-month follow-up period [66]. The Oxford Acute Myocardial Infarction PICSO (OxAMI-PICSO) study enrolled 105 patients with anterior wall STEMI treated with direct PCI. Of these, 25 patients who had an IMR >40 before stenting underwent PICSO, and 50 patients who were not candidates for PICSO had an IMR >40 before stenting. In addition, 30 patients with IMR ≤40 before stent implantation were used as a control group. Postoperatively, no statistically significant difference in IMR was observed between patients who underwent PICSO and controls (*p* = 0.40). However, patients with pre-stenting IMR ≤40 had significantly lower IMR after stenting compared with the PICSO and control groups with initial pre-stenting IMR >40 (*p* = 0.002 and *p* < 0.001, respectively). Moreover, 24–48 h after stent implantation, patients who underwent PICSO had lower IMR compared with controls (24.8 [18.5–35.9] vs. 45.0 [32.0–51.3], *p* < 0.001); at 6 months post-procedure, PICSO patients had lower infarct size compared with controls (26.0% [20.2–30.0] vs. 33.0% [28.0–37.0], *p* = 0.006). These findings indicate that IMR-guided PICSO is feasible for the treatment of anterior wall STEMI, improves microvascular function, and reduces infarct size in patients with STEMI [65]. Several trials on this technology are ongoing, including the US Experimental Device Exemption Trial (PICSO-AMI-II) and a study on the safety and feasibility of PICSO for the treatment of patients with inferior wall STEMI (PICSO-AMI-VNCT 04958421). The results of a recently concluded randomized trial by De Maria *et al*. [66] showed no difference in infarct size reduction between the PICSO-assisted and conventional pPCI groups. Therefore, the results of these ongoing trials are anticipated.

## 5. Issues

Poor quality of life, frequent cardiac visits for investigation, and hospitalization of patients with microvascular disease and refractory angina without the possibility of revascularization may be associated with CMD. In response to this phenomenon, therapies that specifically target and significantly improve CMD are lacking. However, the role of the coronary venous system has long been neglected. In recent years, elective PCI has shown a significant increase in long-term MACE in patients with post-procedural combined coronary microcirculatory disorders [16], leading to new interest in the coronary venous system. The coronary venous system is another avenue for treating coronary microvascular disorders associated with hemodialysis. From the extensive research on the coronary venous system, several therapies have been developed. Ischemic transcatheter CS interventions, mainly consisting of CS reducer implantation and PICSO, can be effective for the treatment of CMD. The field of transcatheter interventions in the CS remains in its nascent stage, with preliminary data demonstrating that modulation of coronary venous pressures is effective in the treatment of refractory patients with microvascular disorders without the possibility of hemodialysis or angina pectoris without the possibility of revascularization. Recent studies suggest that some patients experience minimal or no change in coronary microvascular function after treatment [51, 66]. The reasons for such results may be as follows. First, owing to the heterogeneity of the coronary venous system, the CS has two valves: the Vieussens valve and the Thebesian valve. The Thebesian valve is usually a thin semilunar fold with an open window. However, its morphology varies with some specific structures, such as fibromuscular or muscular, resulting in over 75% of the orifice being covered and lack of an open window, which causes difficulty in CS intubation during cardiac surgery [20, 67, 68]. Alternative venous drainage from the myocardium to the right ventricle (Thebesian venous system) and well-developed alternative CS pathways facilitate venous drainage and prevent redistribution of blood flow to the ischemic myocardium in case of CS occlusion [69, 70]. Second, the size and shape of the CS vary across patients [71]. One study found that CS size was significantly smaller in responders compared with nonresponders (6.6 ± 1.6 mm vs. 8.2 ± 1.4 mm, respectively; *p* = 0.04) [72]. Currently, CS dimensions are not routinely assessed before resetter implantation. If subsequent studies can confirm that CS size is the cause of non-response, assessment of CS size before implantation should be considered. Zivelonghi reported in his case study that 43 patients underwent implantation of CS tapering tubes, and five patients were nonresponsive at 6 months of follow-up. CS angiography demonstrated a free flow of contrast through the struts of the tapering tubes, and retrograde pressure recordings performed in the CS did not show any pressure gradient in the neck of the device, suggesting incomplete endothelialization. Thus, incomplete device endothelialization may partially explain the lack of a clinical response in some patients [73]. Any extremes in CS sizing may make instrument delivery difficult and lead to poor instrument endothelialization. It is recommended that the instrument size exceed the CS by 10%–20% relative to the CS to prevent instrument displacement and promote injury-induced tissue growth activation, which enhances subsequent instrument endothelialization [71]. Fourth, different CAD “phenotypes” (i.e., chronic total occlusion (CTO), diffuse disease, microvascular angina, and high-risk single- or double-branch vascular disease) respond differently to tapering tube implantation. Decelerators may function only for a limited time; however, symptomatic recurrence may occur due to CAD progression, particularly in patients with complex CAD, who are often at higher risk of progression. Therefore, when patients present with angina recurrence, their coronary anatomy should be re-evaluated using coronary angiography to check for disease progression. If there is no significant progression of CAD, repeat ischemic testing should be considered to determine the possibility of new microvascular dysfunction [74]. In addition, the presence of epicardial or microvascular myocardial ischemia, as determined by dobutamine loading echocardiography, single-photon emission computed tomography, or loading CMR, is necessary prior to considering decelerator implantation; however, there is no set ischemic threshold. The improvement in myocardial perfusion observed after reducer therapy was significantly greater in myocardial segments with elevated baseline ischemia, as assessed using load CMR. Conversely, patients with small ischemic areas did not show significant changes [75]. Further studies are needed to identify and validate the minimum ischemia threshold that can differentiate between low and high probabilities of treatment response.

## 6. Conclusion

CMD is often accompanied by epicardial disease with poor outcomes despite successful revascularization and OMT. Therefore, new studies have been conducted on the treatment of patients with CMD, with the primary goal of reducing disabling symptoms and improving patients’ quality of life through new therapies. In recent years, research on the coronary venous system has gradually increased, and data from these studies suggest that CS reducer and PICSO interventions can significantly alleviate clinical symptoms in 70%–80% of patients with refractory angina pectoris and acute myocardial infarction who suffer from microvascular disease without the possibility of hemodialysis. These interventions act by regulating coronary venous pressure. However, findings from a recent randomized trial demonstrated no difference in infarct size reduction between the PICSO-assisted and conventional pPCI groups. In this study, only half of the enrolled patients received PICSO therapy within the recommended optimal duration of 45 minutes, suggesting that insufficient treatment exposure may have limited the therapeutic efficacy of PICSO. Given that research on this approach is still in its infancy, larger cohort studies are required to further evaluate and improve these treatments.
